# Supplementary material for: Production of Terretonin N and Butyrolactone I by Thermophilic Aspergillus terreus TM8 Promoted Apoptosis and Cell Death in Human Prostate and Ovarian Cancer Cells
Source: Molecules. 2021 May 10;26(9):2816. doi: 10.3390/molecules26092816 (PMC8126039; doi:10.3390/molecules26092816)
Supplement: Supplementary file 1 [file molecules-26-02816-s001.zip › molecules-1196416-supplementary.pdf]

Supporting Information

Production of Terretonin N and Butyrolactone I by Thermophilic *Aspergillus terreus* TM8 promoted apoptosis and cell death in human prostate and ovarian cancer cells

Ayman A. Ghfar[1*], Mohammad Magdy El-Metwally[2], Mohamed Shaaban[3], Sami A. Gabr[4], Nada S. Gabr[5], Marwa S. M. Diab[6], Ahmad Aqel[1], Mohamed A. Habila[1], Wahidah H. Al-Qahtani[7], Mohamed Y. Alfaifi[8], Serag Eldin I. Elbehairi[8,9,*], Bayan Ahmed AlJumah[10]

[1] Advanced material Research Chair, Chemistry Department P. O. Box 2455, College of Science, King Saud University, Riyadh 11451, Saudi Arabia.

[2] Botany and Microbiology Department, Faculty of Science, Damanhour University, Damanhour, Egypt.

[3] Chemistry of Natural Compounds Department, Division of Pharmaceutical Industries, National Research Centre, El-Behoos St. 33, Dokki-Cairo 12622, Egypt

[4] Department of Anatomy, Faculty of Medicine, Mansoura University, Mansoura, Egypt

[5] Faculty of Medicine, Mansoura University, Mansoura, Egypt

[6] Molecular Drug Evaluation Department, National Organization for Drug Control & Research (NODCAR), Giza 12553, Egypt

[7] Department of Food Sciences & Nutrition, College of Food & Agriculture Sciences, King Saud University, Riyadh, Saudi Arabia

[8] Biology Department, Faculty of Science, King Khalid University, Abha 9004, Saudi Arabia.

[9] Cell Culture Lab, Egyptian Organization for Biological Products and Vaccines (VACSERA Holding Company), 51 Wezaret El-Zeraa St., Agouza, Giza, Egypt.

[10] Department of Pharmaceutical Practice, College of Pharmacy, Princess Nourahbint Abdulrahman University, Riyadh 11671, Saudi Arabia

* Correspondence: Ayman Ghfar, Chemistry Department, College of Science, King Saud University, Riyadh-11451, Kingdom of Saudi Arabia. Tel: + 966545757290, E-mail: aymanghfar@gmail.com; Serag Eldin I. Elbehairi, Biology Department, Faculty of Science, King Khalid University, Abha 9004, Saudi Arabia. E-mail: seragalone@gmail.com.

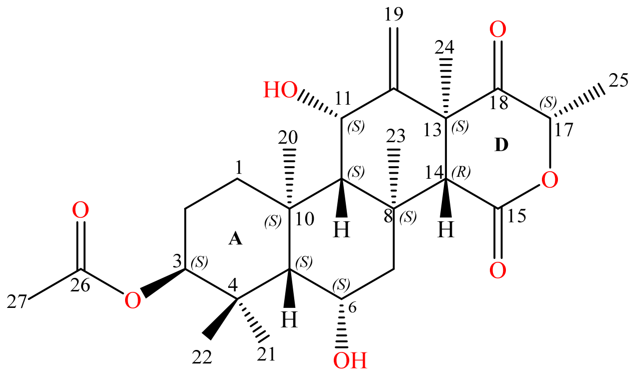

1

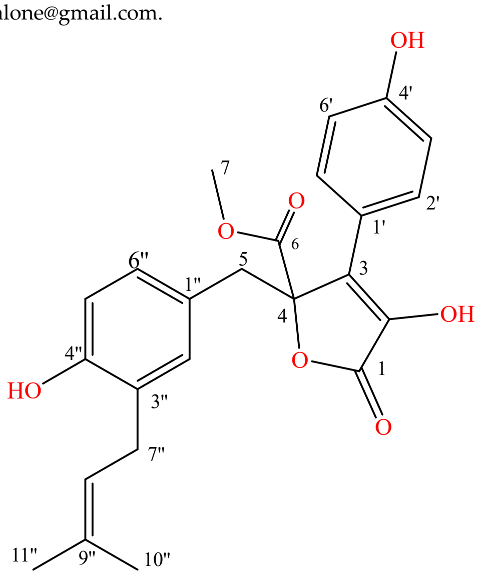

2

**Table of Contents:**	**Page**
Physico-properties of terretonin N **(2)** and butyrolactone I **(2)**	S3
**Figure S1-S10:** MS and NMR spectral data of terretonin N (**1**)	S4-S13
**Figure S11:** COSY ( ) and key HMBC ( ) correlations of terretonin N (**1**)	S14
**Table S1:** ^{13}C (125 MHz) and ^{1}H (500 MHz) NMR data of terretonin N (**1**) in $CDCl_3$	S14
**Figure S12-S17:** MS and NMR spectral data of butyrolactone I (**2**)	S15-S20
**Figure S18**: H,H **COSY** ( ) and selected HMBC ( ) correlations of butyrolactone I (**2**)	S21
**Table S2**: ^{13}C (125 MHz) and ^{1}H (500 MHz) NMR data of butyrolactone I (**2**) in Acetone-d_6:	S21

Physico-properties of terretonin N (2) and butyrolactone I (2)

Terretonin N (1)

$C_{26}H_{38}O_7$ (462), colorless solid, $\boldsymbol{R}_f$ = 0.54 (CH_2Cl_2/ 5% MeOH), UV non absorbing-absorbing (254 nm) or fluorescence, pink and later as violet staining with anisaldehyde/sulfuric acid. $[\alpha]_D^{20}$ 114 (*c*= 0.1, MeOH). **^{1}H NMR** (500 MHz, $CDCl_3$, see Table S1), **^{13}C NMR** (125 MHz, $CDCl_3$, see Table S1). **(+)-ESI MS**: *m/z* (%) = 485 ($[M+Na]^+$, 100), 947 ($[2M+Na]^+$, 0.58). **(-)-ESI MS**: *m/z* (%) = 461 ($[M-H]^-$,62), 891 ($[2M-H]^-$, 4), **(-)-ESI-HRMS**: *m/z* = 461.2547 $[M-H]^+$ (Calc. 461.2545 for $C_{26}H_{37}O_7$).

Butyrolactone I (2)

$C_{24}H_{24}O_7$ (424), colorless solid, $\boldsymbol{R}_f$ = 0.43 (CH_2Cl_2/ 7% MeOH), UV absorbing (254 nm), intense violet staining with anisaldehyde/sulfuric acid. **^{1}H NMR** (500 MHz, Acetone-d_6), **^{13}C NMR** (125 MHz, Acetone-d_6), see Table (**3**). **(+)-ESI MS**: *m/z* (%) = 447 ($[M+Na]^+$, 81), 871 ($[2M+Na]^+$, 100). **(-)-ESI MS**: *m/z* (%) = 423 ($[M-H]^-$,13), 847 ($[2M-H]^-$, 4), **(+)-ESI-HRMS**: *m/z* = 447.1414 $[M+Na]^+$ (Calc. 447.1414 for $C_{24}H_{24}NaO_7$), **(-)-ESI-HRMS**: *m/z* = 423.1435 $[M-H]^-$ (calc. 423.1449 for $C_{24}H_{23}O_7$).

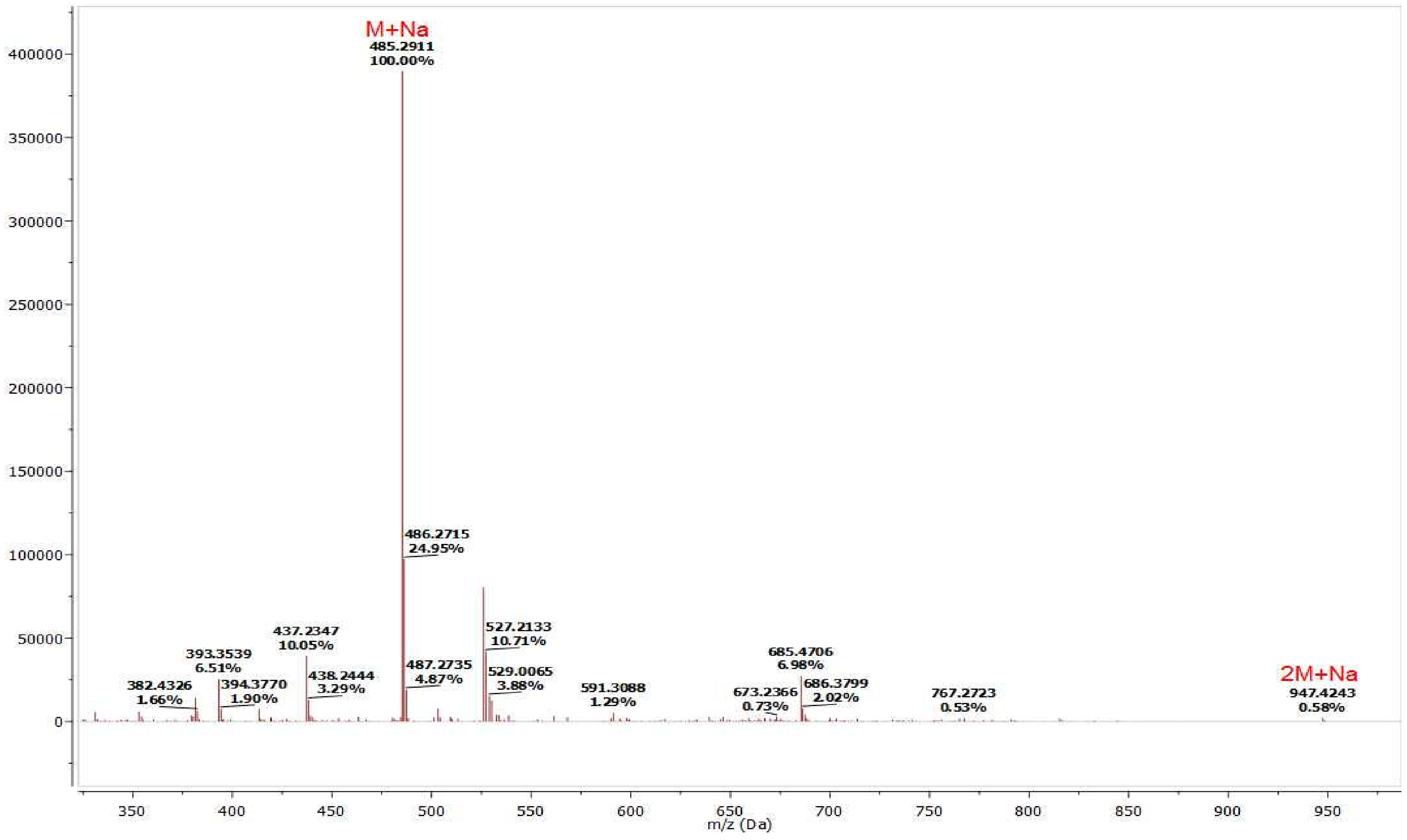

Figure S1: (+)-ESIMS of terretonin N (**1**)

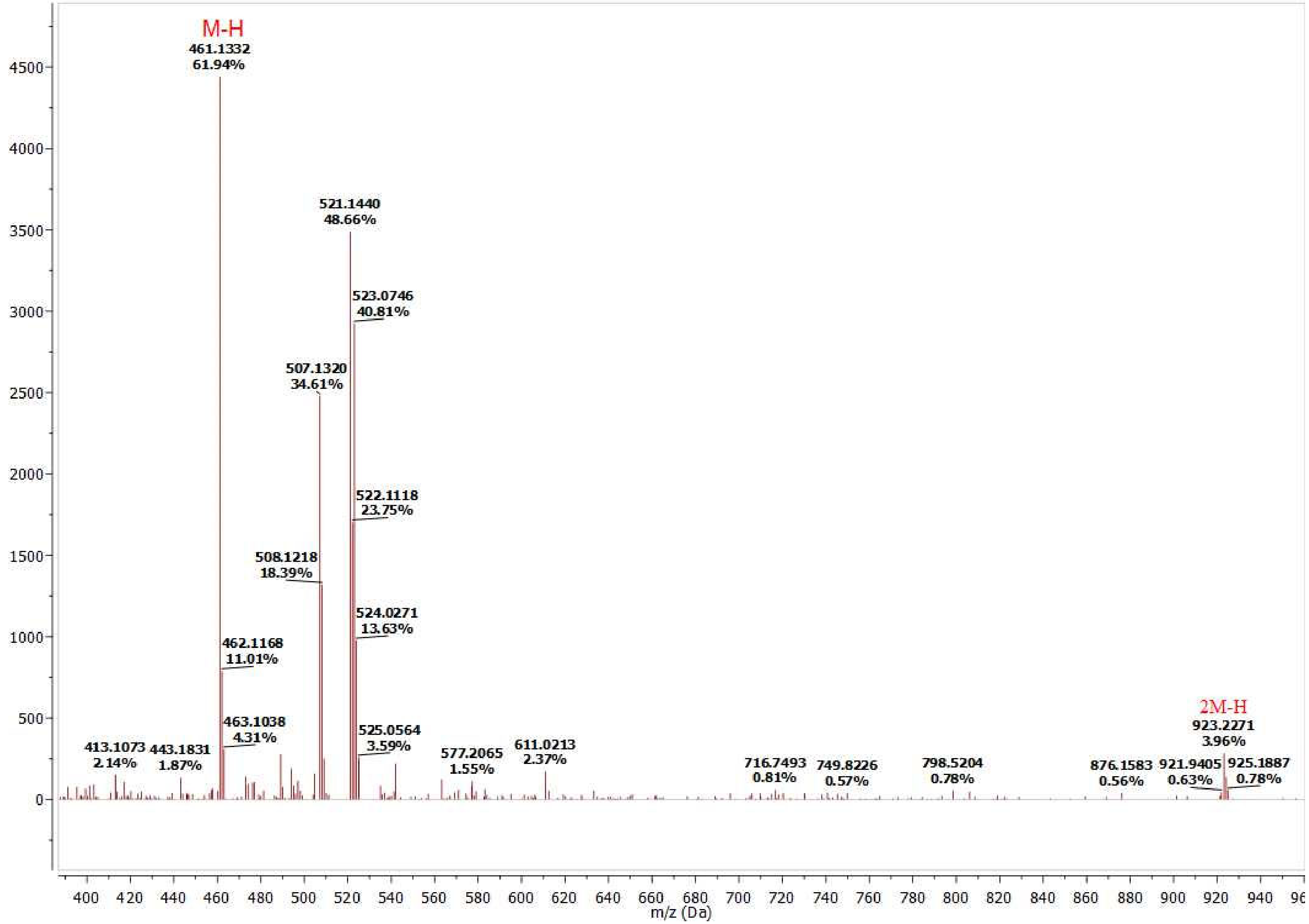

Figure S2: (-)-ESIMS of terretonin N (**1**)

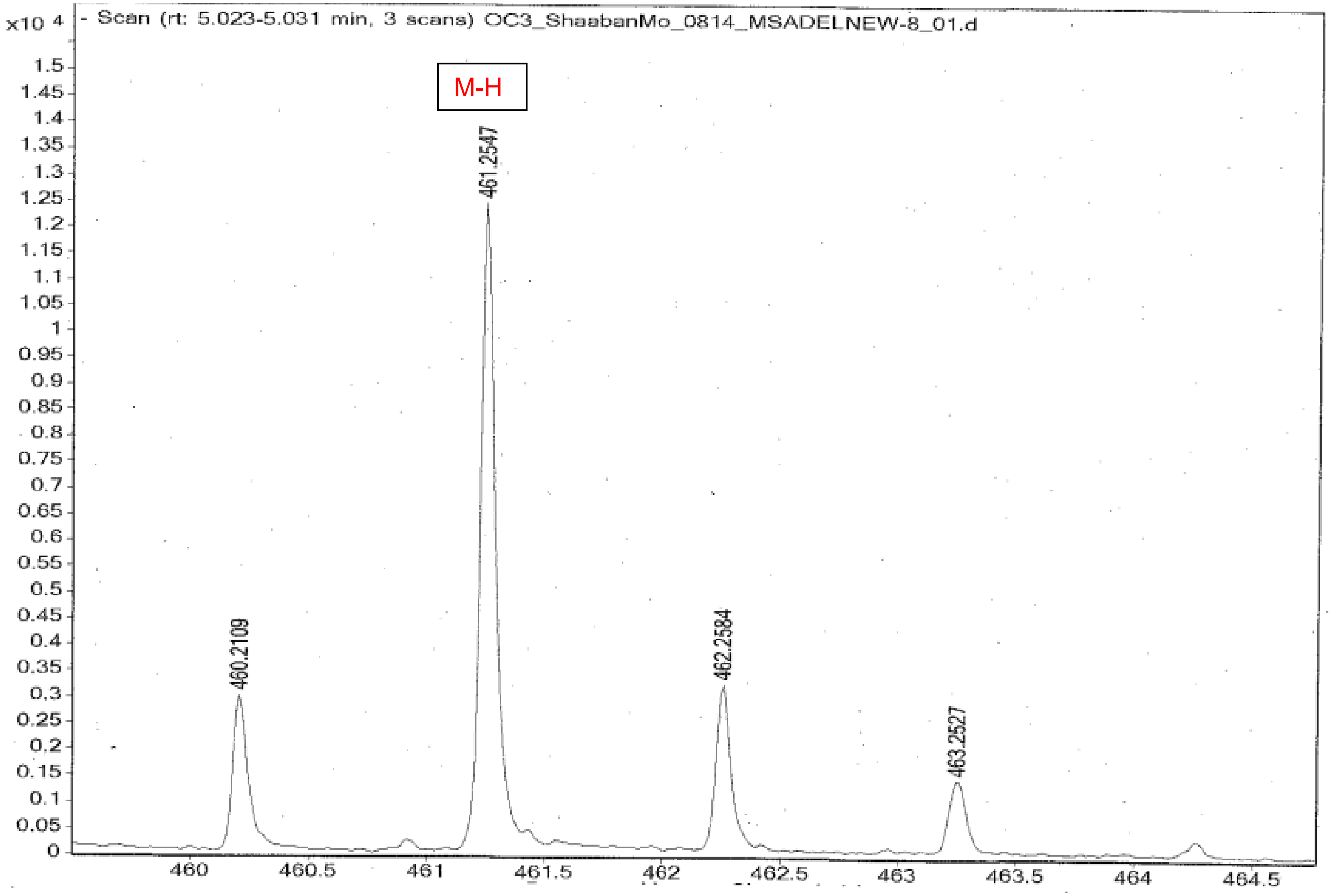

Figure S3: HR-ESI MS of terretonin N (**1**)

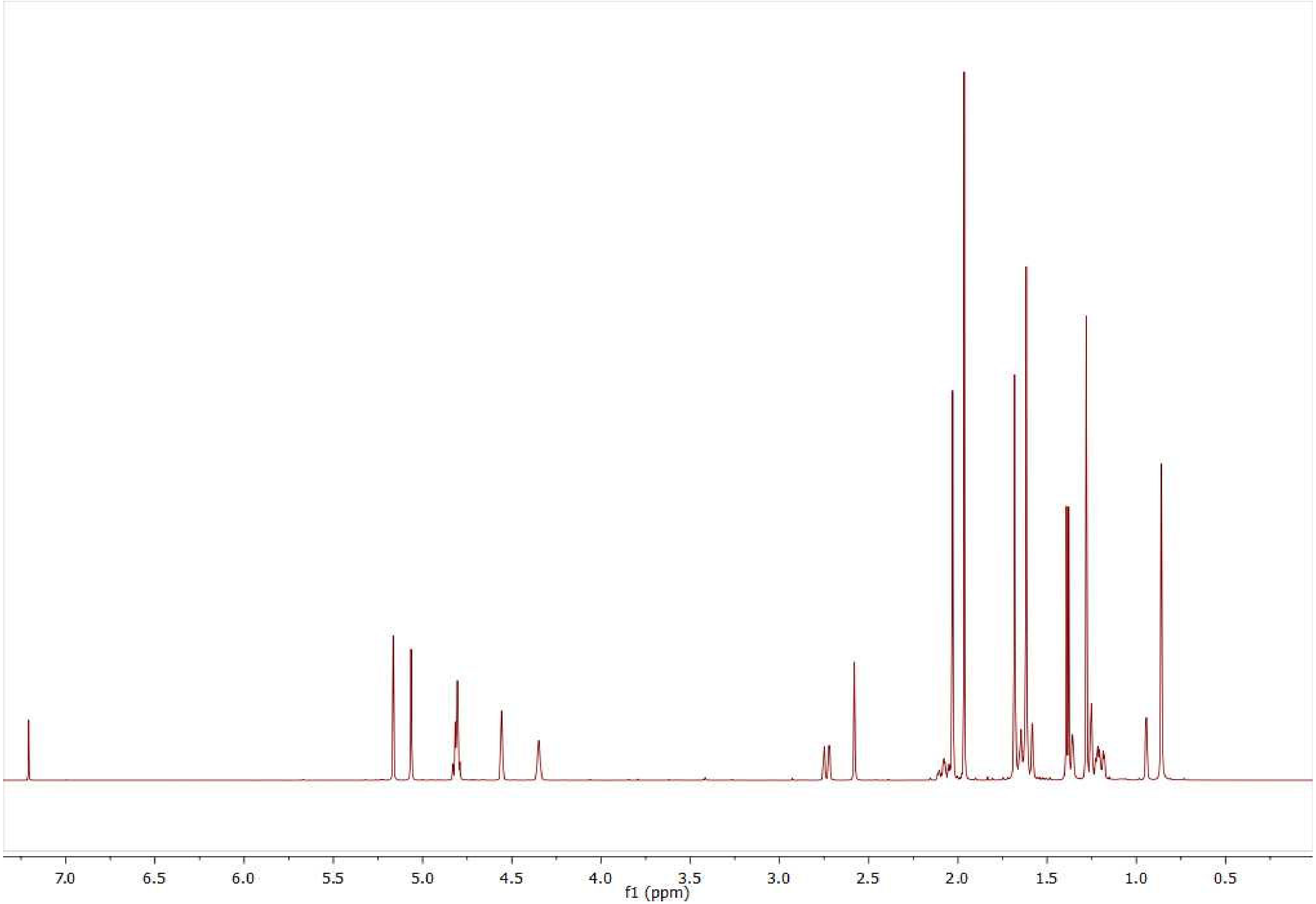

Figure S4: ^{1}H NMR spectrum ($CDCl_3$, 500 MHz) of terretonin N (**1**)

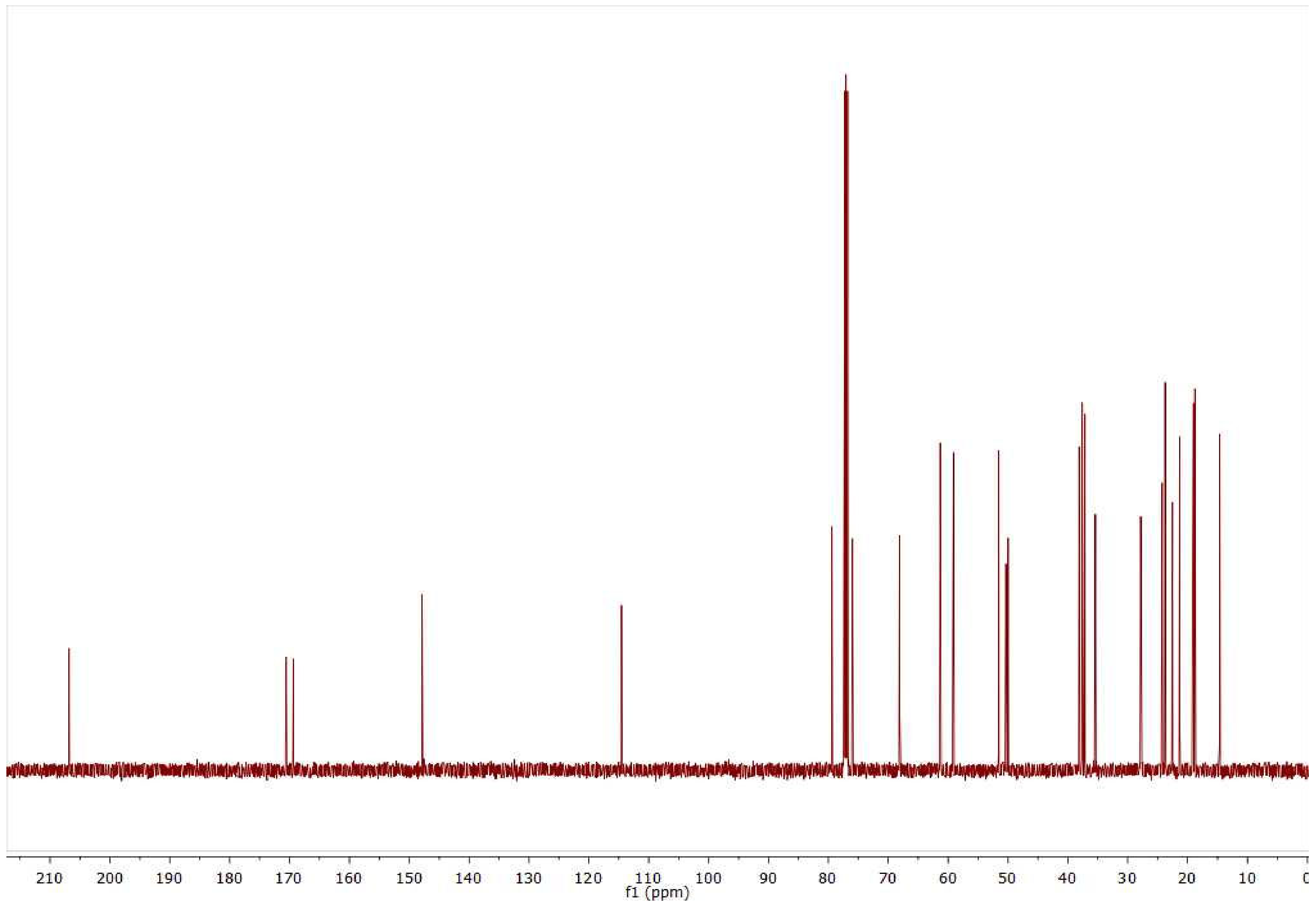

Figure S5: ^{13}C NMR spectrum ($CDCl_3$, 125MHz) of terretonin N (**1**)

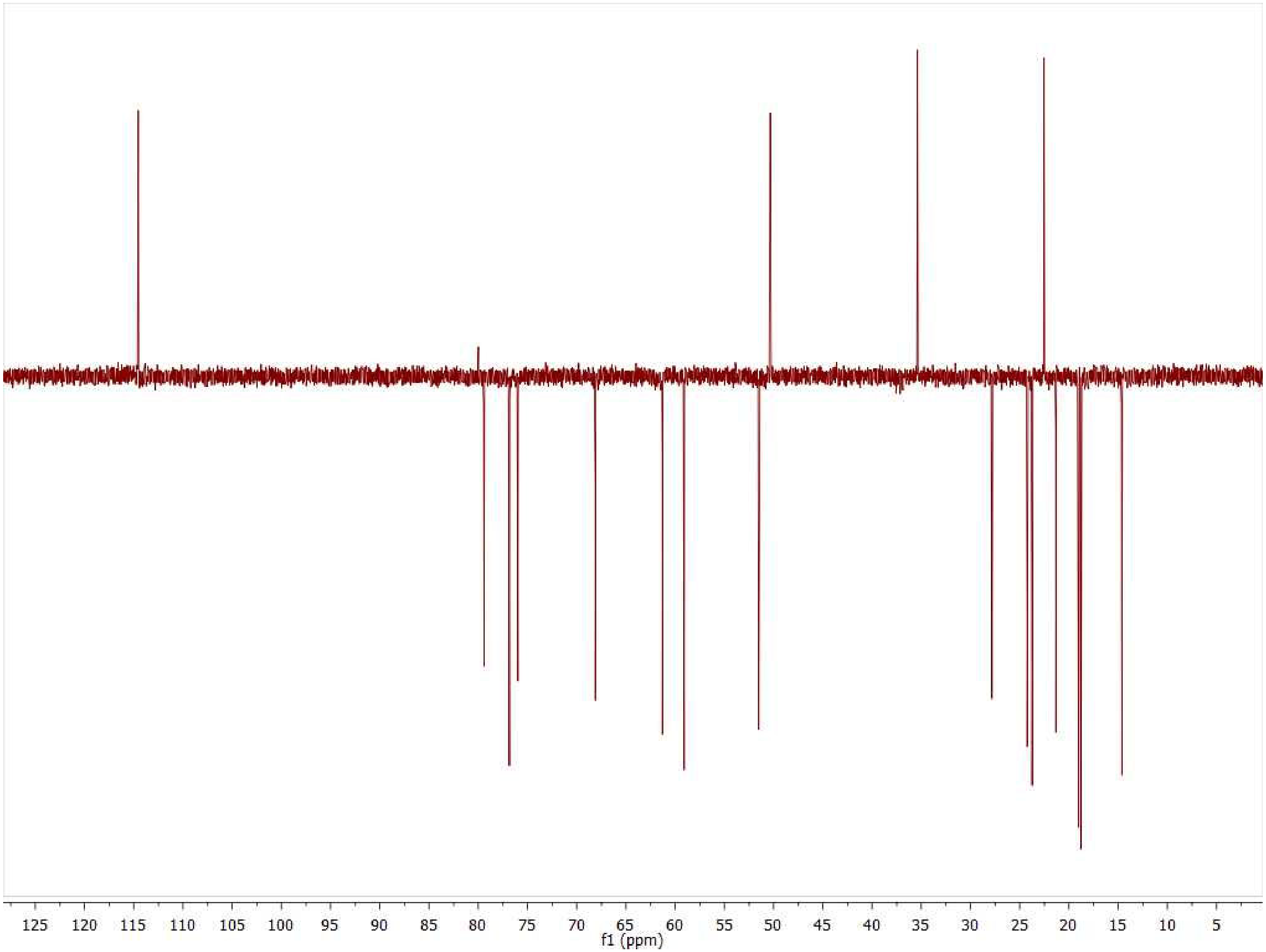

Figure S6: DEPT spectrum ($CDCl_3$, 125 MHz) of terretonin N (**1**)

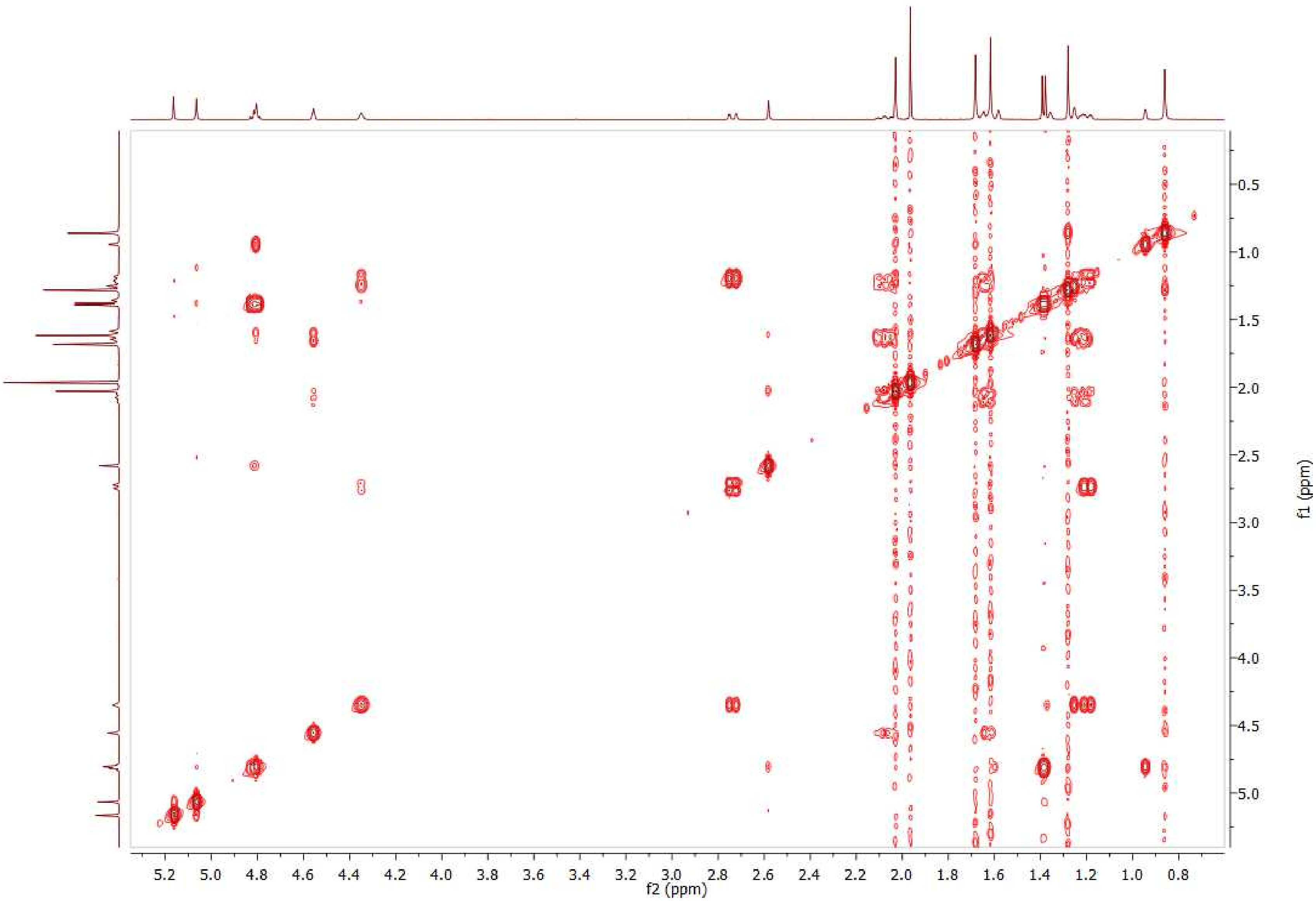

Figure S7: H,H-COSY spectrum ($CDCl_3$, 500 MHz) of terretonin N (**1**)

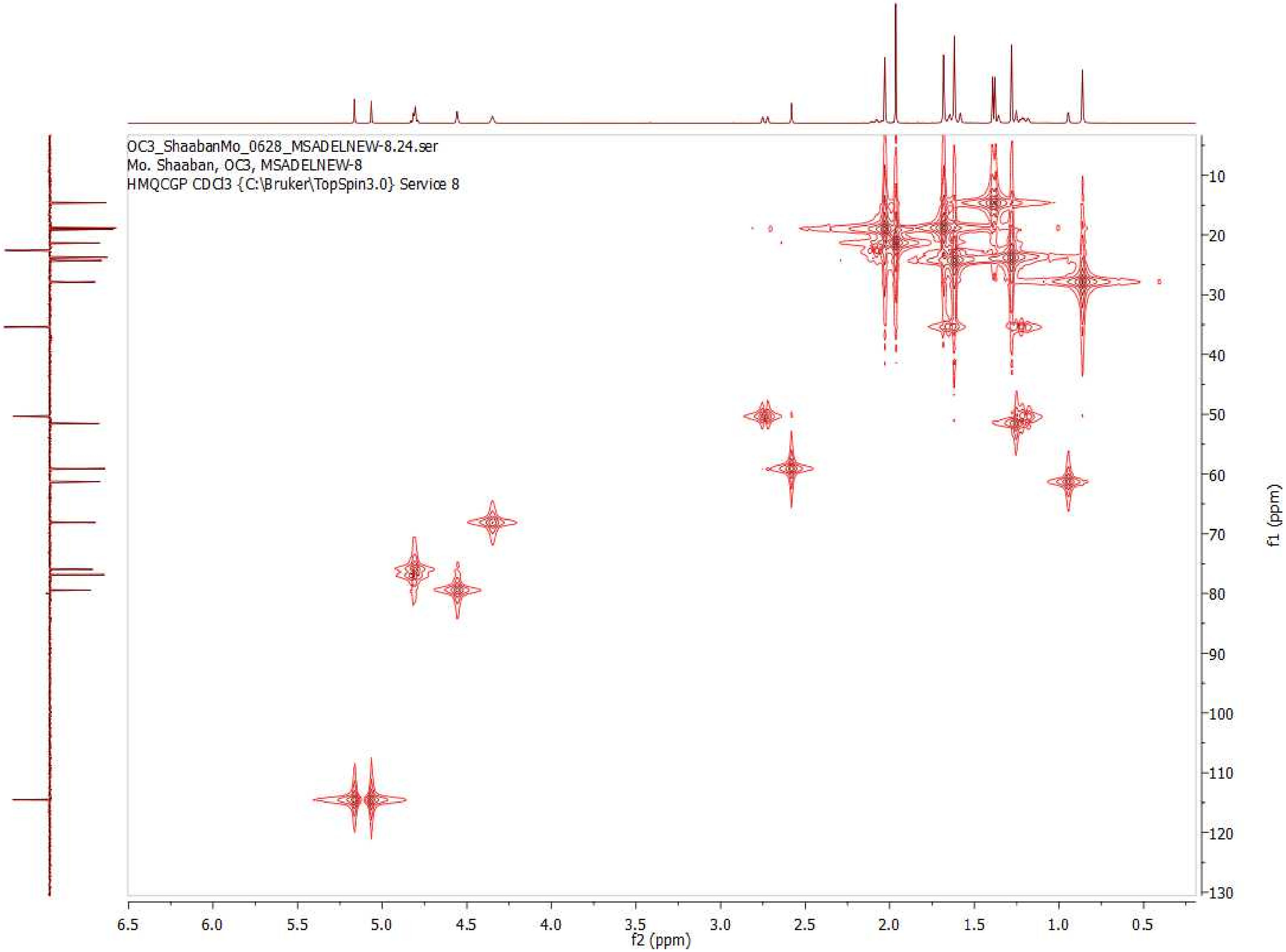

Figure S8: HMQC spectrum ($CDCl_3$, 500 MHz) of terretonin N (**1**)

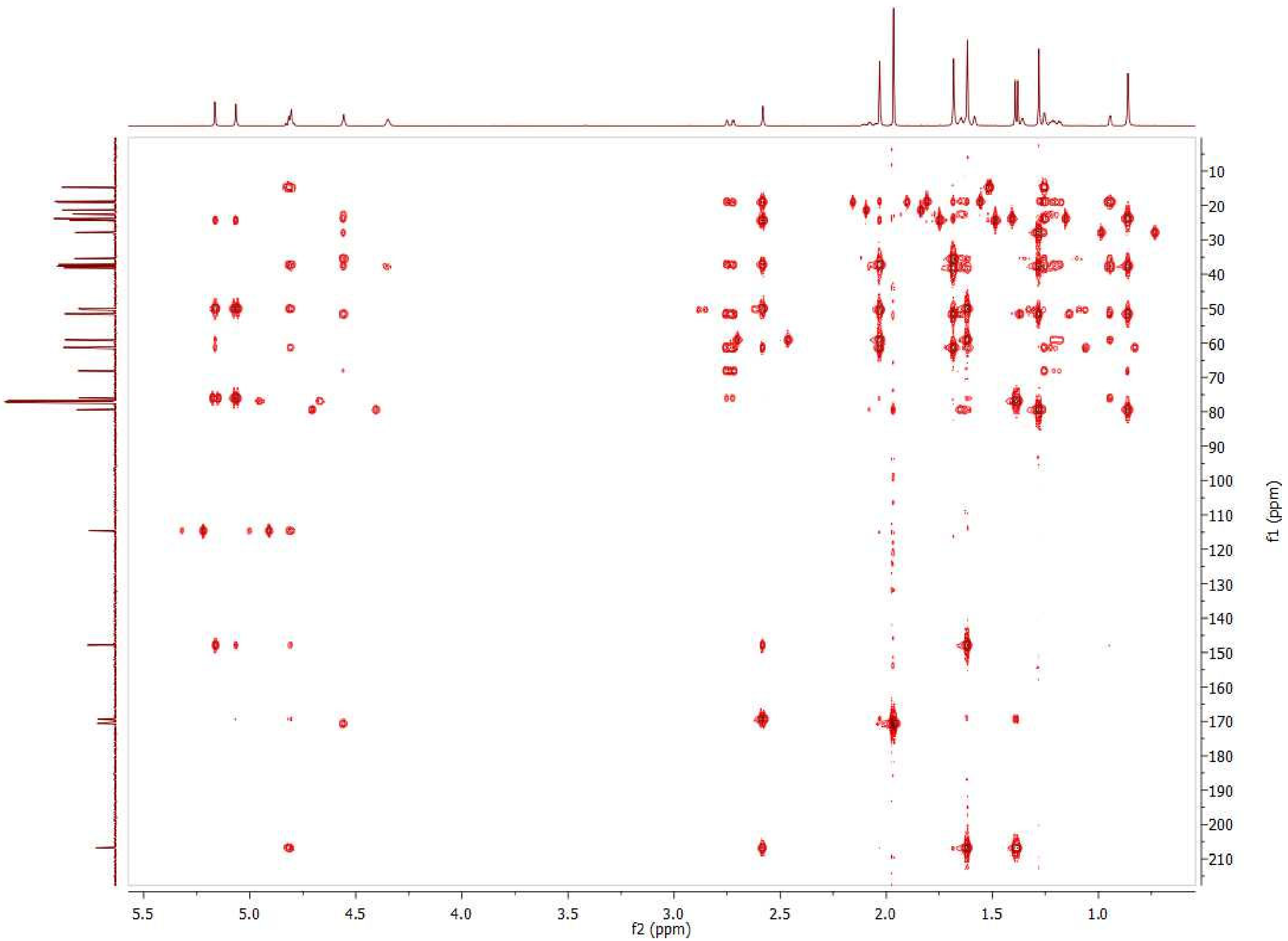

Figure S9: HMBC spectrum ($CDCl_3$, 500 MHz) of terretonin N (**1**)

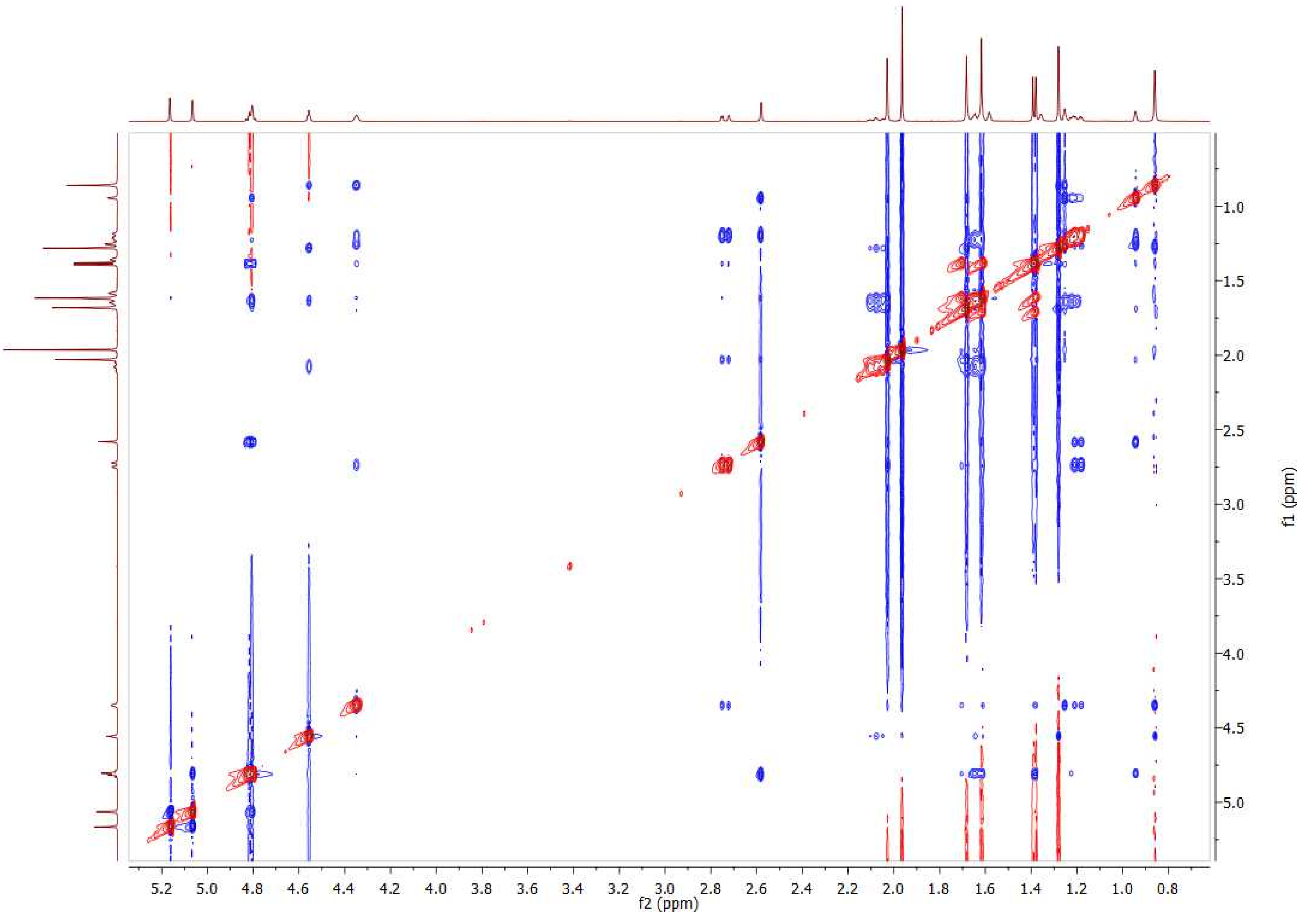

Figure S10: NOESY spectrum ($CDCl_3$, 500 MHz) of terretonin N (**1**)

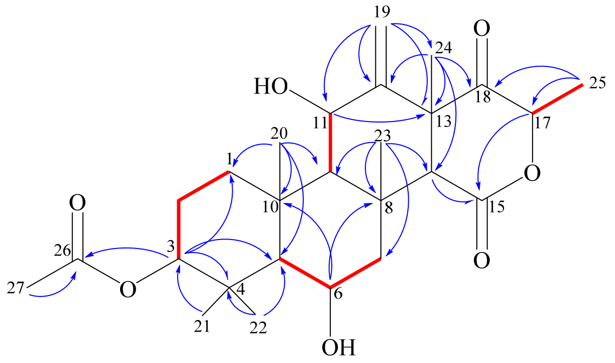

Figure S11. COSY (▬) and key HMBC (⌒) correlations of terretonin N (**1**)

Table S1: ^{13}C (125 MHz) and ^{1}H (500 MHz) NMR data of terretonin N (**1**) in $CDCl_3$

Position	δ_C [ppm]	δ_H [ppm] (m, *J* [Hz])	Position	δ_C [ppm]	δ_H [ppm] (m, *J* [Hz])
1	35.4	1.65 (m), 1.21 (m)	14	59.1	2.58 (s)
2	22.5	1.95 (m), 1.65 (m)	15	169.4	-
3	79.4	4.55 (t, 3.0)	17	76.8	4.81 (m)
4	37.6	-	18	206.8	-
5	51.5	1.25 (m)	19	114.5	5.16 (s), 5.06 (s)
6	68.1	4.35 (t, 3.0)	20	18.8	1.68 (s)
7	50.3	2.74 (dd, 14.5, 2.7), 1.19 (m)	21	27.8	0.86 (s)
8	37.1	-	22	23.7	1.28 (s)
9	61.3	0.95 (d, 2.4)	23	19.0	2.03 (s)
10	38.1	-	24	24.2	1.62 (s)
11	76.0	4.81 (m)	25	14.6	1.38 (d, 6.4)
12	147.9	-	26	170.6	-
13	50.0	-	27	21.3	1.96 (s)

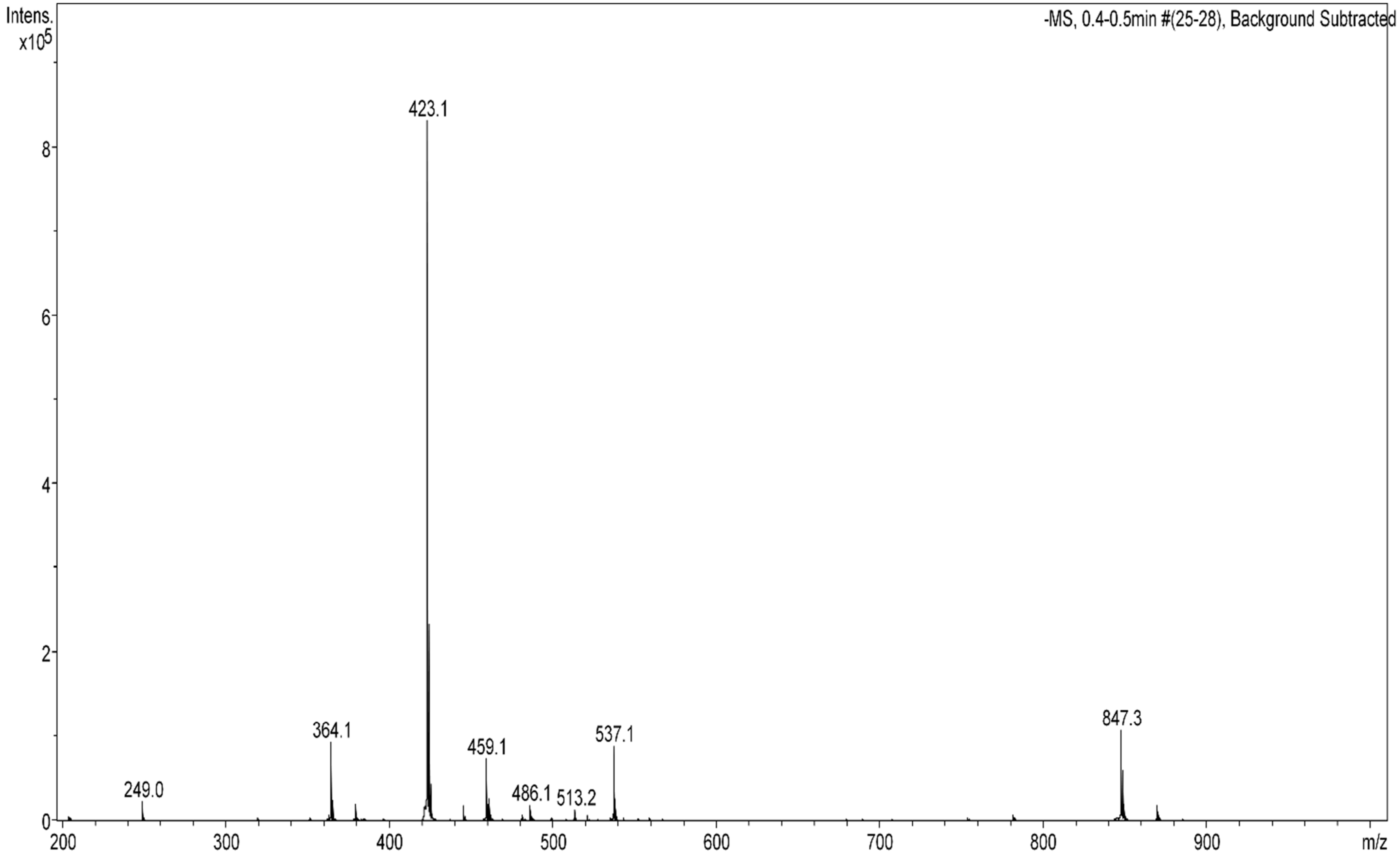

Figure S12: (-)-ESIMS of butyrolactone I (**2**)

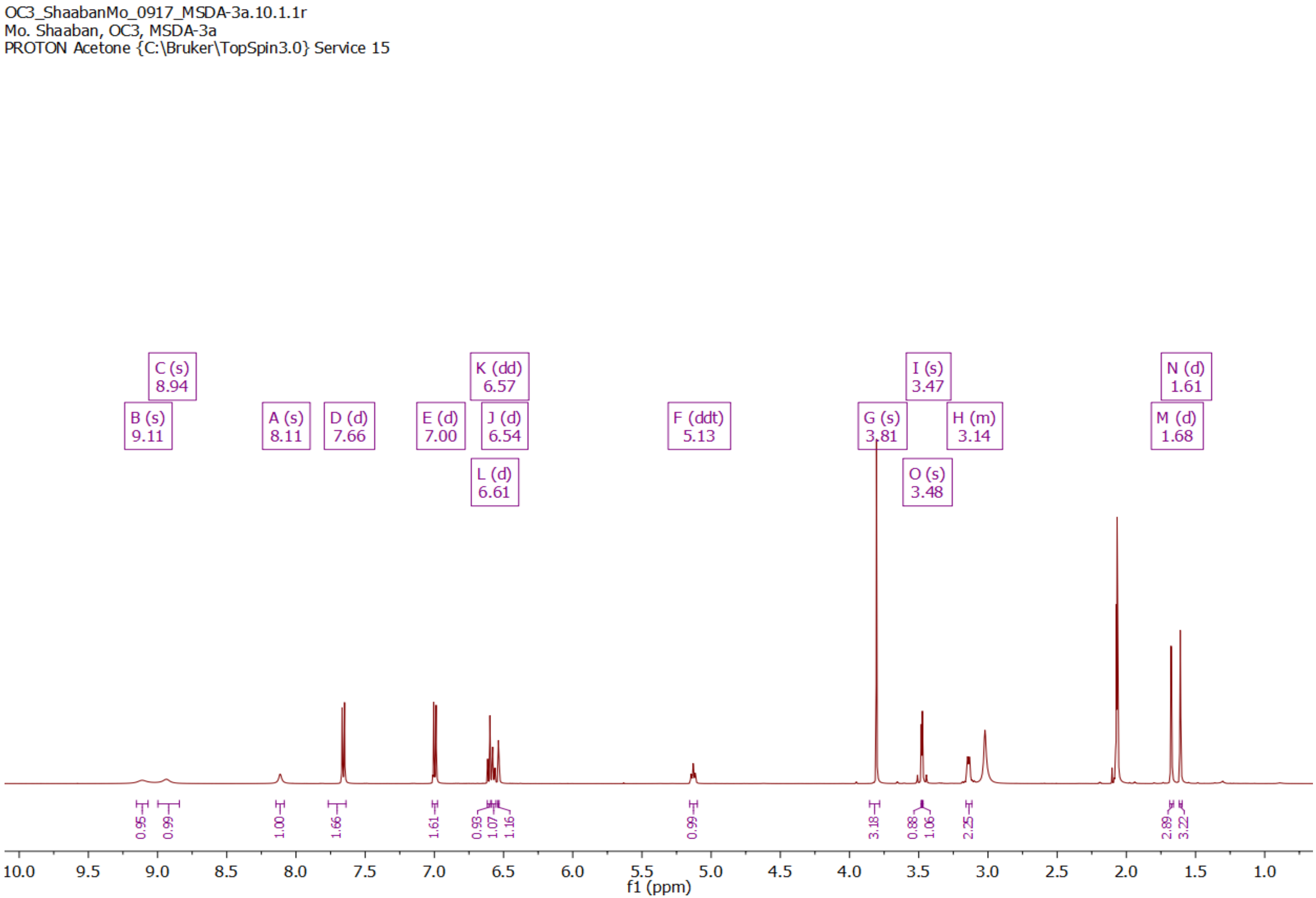

Figure S13: ^{1}H NMR (500 MHz, Acetone-d_6) of butyrolactone I (**2**)

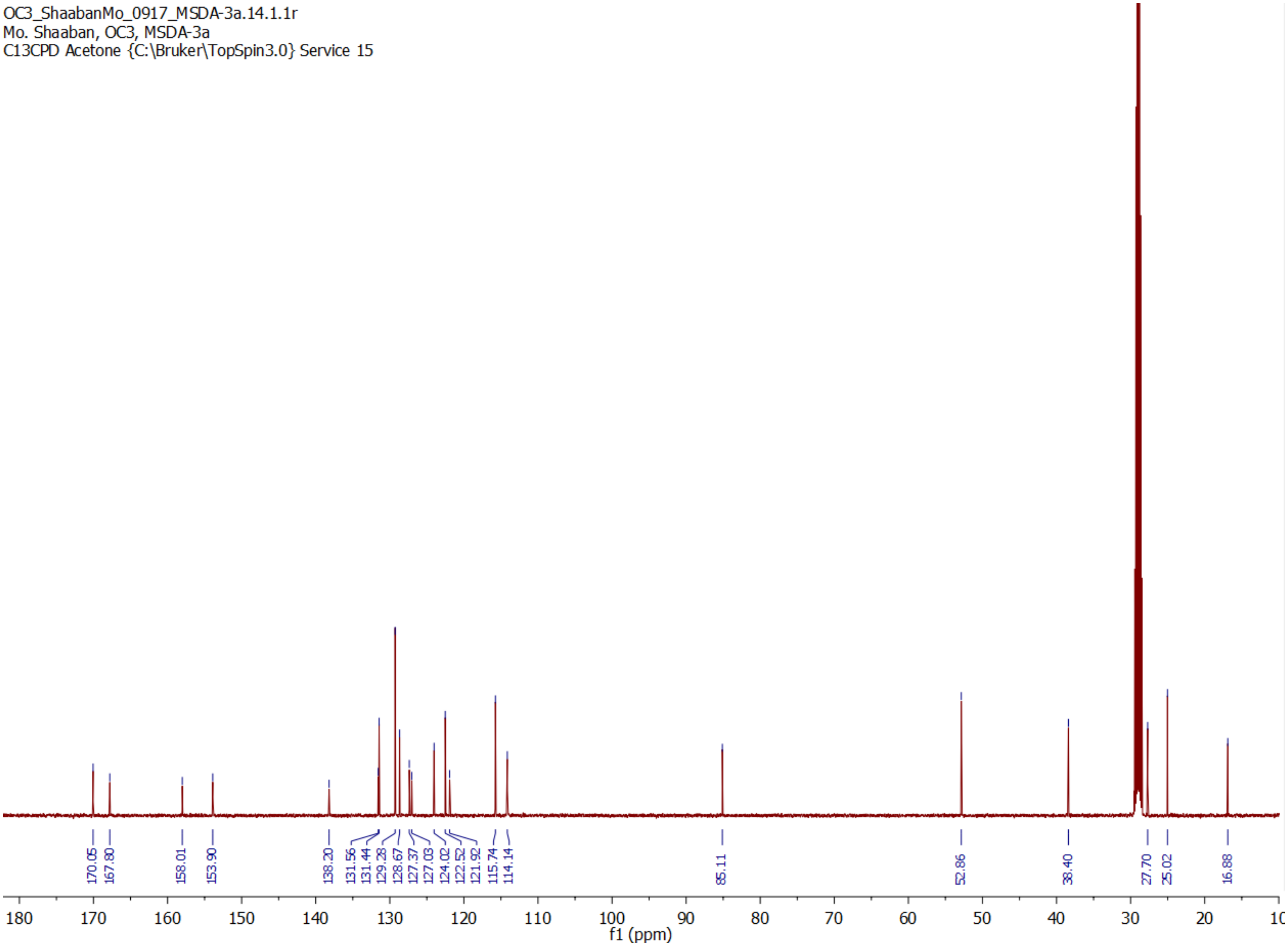

Figure S14: ^{13}C NMR (125 MHz, Acetone-d_6) of butyrolactone I (**2**)

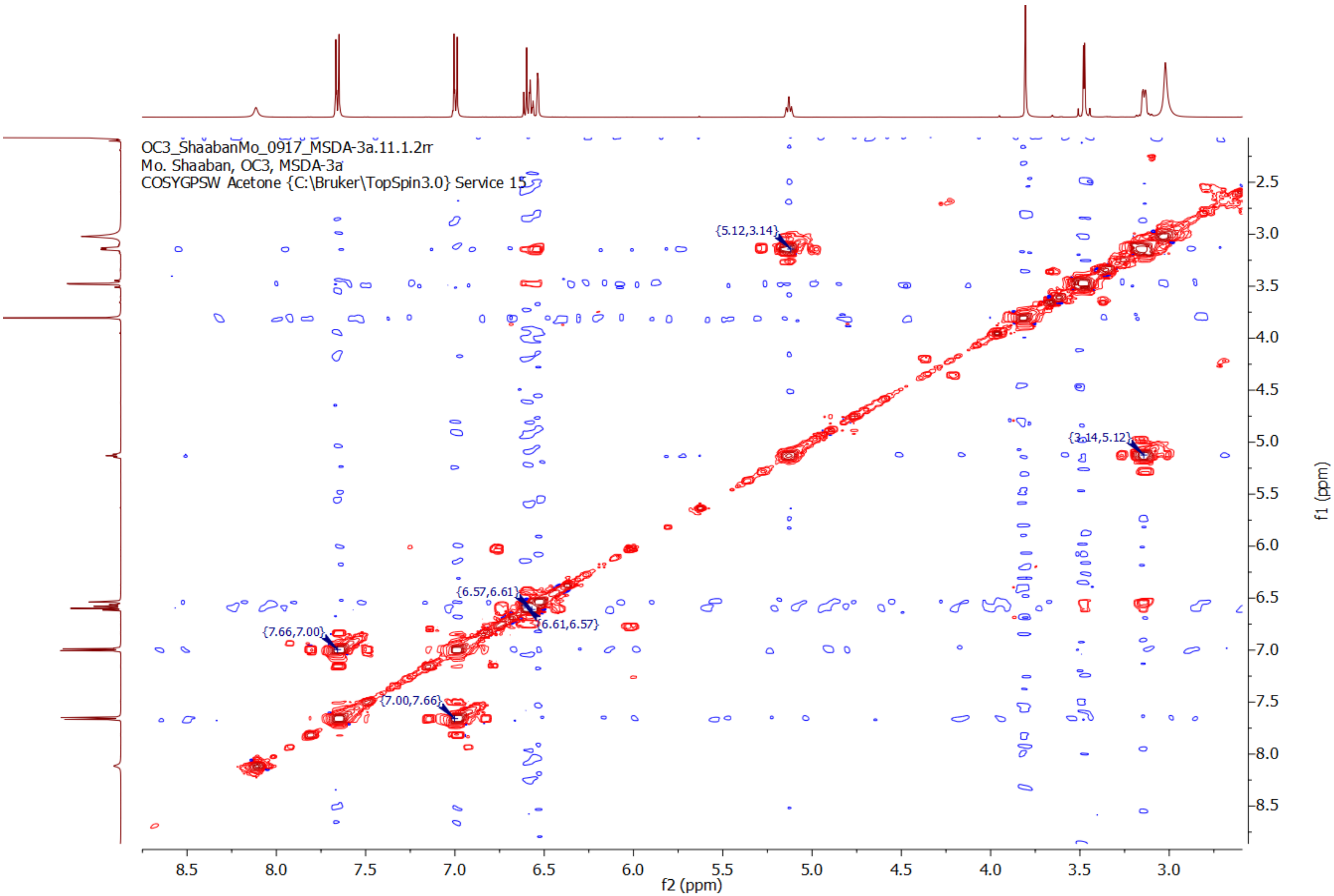

Figure S15: H-H-COSY spectrum of butyrolactone I (**2**)

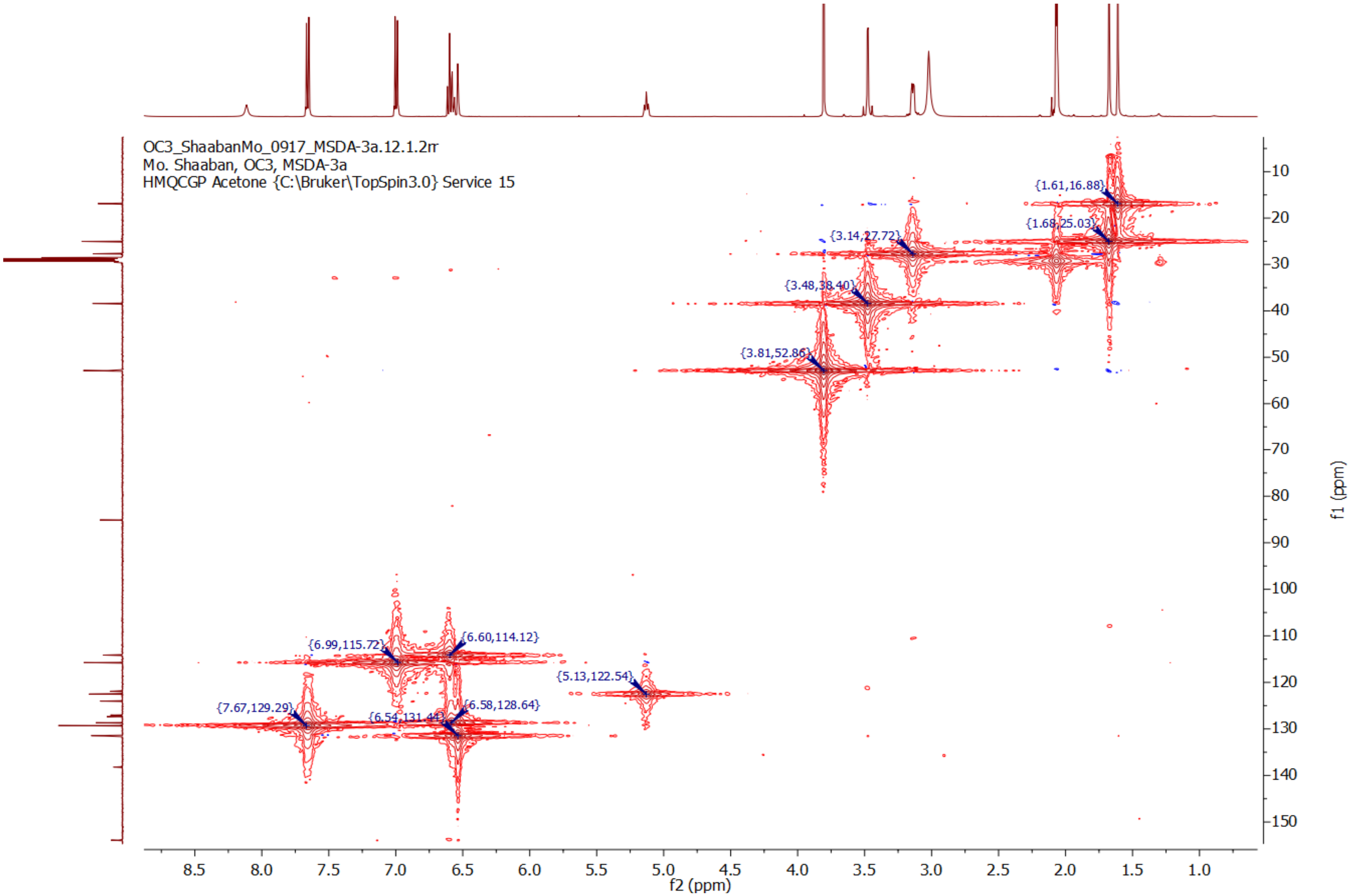

Figure S16: HMQC spectrum of butyrolactone I (**2**)

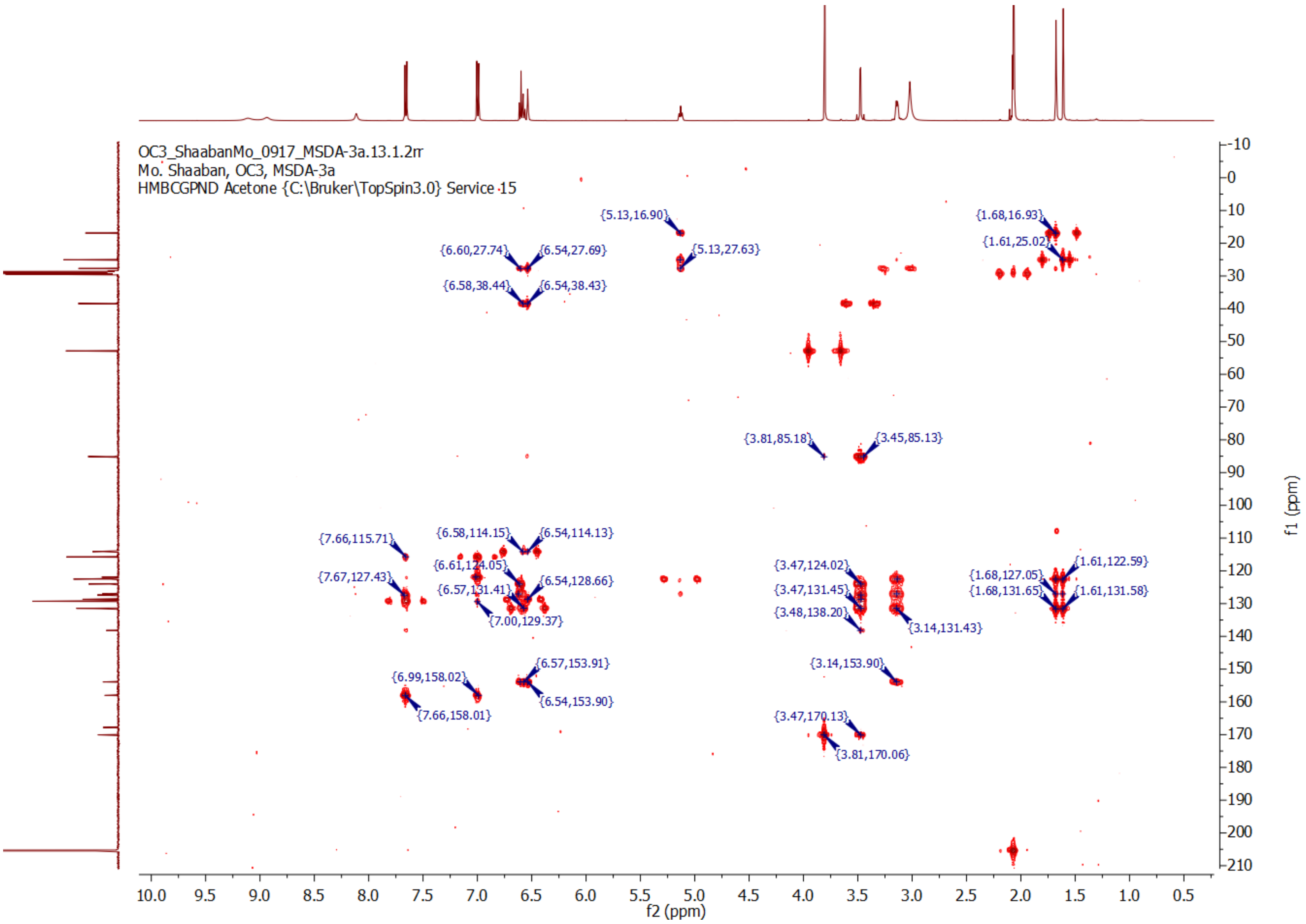

Figure S17: HMBC spectrum of butyrolactone I (**2**)

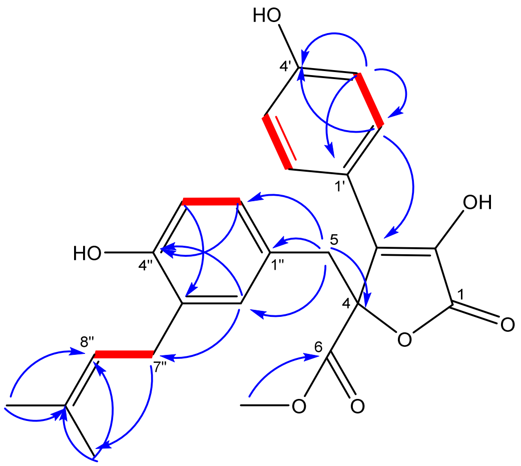

Figure S18: H,H **COSY** ( ) and selected HMBC ( ) correlations of butyrolactone I (**2**)

Table S2: ^{13}C (125 MHz) and ^{1}H (500 MHz) NMR data of butyrolactone I (**2**) in Acetone-d_6:

**Position**	**δ_C (ppm)**	**δ_H (*J* in Hz)**	**position**	**δ_C (ppm)**	**δ_H (*J* in Hz)**
1	167.8	-	1"	124.0	-
2	138.2	-	2"	131.4	6.54 (d,2.2)
3	127.4	-	3"	127	-
4	85.1	-	4"	153.9	-
5	38.4	3.48 (s), 3.47 (s)	5"	114.1	6.61(d,8.1)
6	170.1	-	6"	128.7	6.57(dd,8.2,2.2)
7	52.9	3.81 (s)	7"	27.7	3.14(m)
1'	121.9	-	8"	122.5	5.13(ddt,8.8,5.9,1.5)
2',6'	129.3	7.66(d,8.9)	9"	131.6	-
3',5'	115.7	7.0(d,8.8)	10"	25.0	1.68(d,1.4)
4'	158.0	-	11"	16.9	1.61(d,1.2)
